# Dietary Patterns and Poor Semen Quality Risk in Men: A Cross-Sectional Study

**DOI:** 10.3390/nu10091162

**Published:** 2018-08-24

**Authors:** Anna Danielewicz, Katarzyna Eufemia Przybyłowicz, Mariusz Przybyłowicz

**Affiliations:** 1 Department of Human Nutrition, University of Warmia and Mazury in Olsztyn, ul. Słoneczna 45F, 10-718 Olsztyn, Poland; katarzyna.przybylowicz@uwm.edu.pl; 2 Center of Gynecology, Endocrinology and Reproductive Medicine Artemida in Olsztyn, Jagiellońska 78, 10-229 Olsztyn, Poland; mprzybylowicz@wp.pl

**Keywords:** dietary patterns, western diet, sperm quality, male fertility, BMI

## Abstract

The etiology of diminished sperm quality in about 30% of male infertility cases generally remains unexplained. Some studies have suggested that specific nutritional factors can affect semen quality. The aim of this study was to evaluate an association between dietary patterns (DPs) and the risk of abnormal semen quality parameters in men. This cross-sectional study was carried out in 114 men aged 20–55 years from Poland. Semen parameters were assessed via computer-aided semen. Diet was assessed by a food frequency questionnaire (FFQ). DPs were derived using Principal Component Analysis (PCA). Two DPs were derived: Pro-healthy and Western. After adjusting for potential confounders, the risk of abnormal progressive motility was significantly higher in the middle (OR: 2.89, 95% CI: 1.03–8.09) and upper (OR: 7.78, 95% CI: 1.52–15.06) tertiles of the Western DP. A trend for increased risk of the abnormal total count, progressive motility, and morphology (*P*-trend < 0.050) was found in Western DP. To conclude, the Western DP may increase the risk of abnormal semen parameters, whereas no association was found in the case of Pro-healthy DP. These findings stand in contrast to an increasing number of research findings indicating a positive relation between intake of healthy foods or diet and semen quality parameters. The results highlight the need to study whether modifications in diet and lifestyle factors improve semen quality.

## 1. Introduction

Over the past decades, several studies have provided evidence that semen quality in humans is decreasing, which might lead to an increase in male subfertility [1]. It is estimated that the infertility problem concerns 48.5 million couples on a global scale, but its occurrence and etiology are varied in individual countries [2]. The highest ratio of infertility caused by a male factor was found in Central and Eastern Europe, where it is estimated that 8–12% of men are infertile, which in the case that 20% of pairs are infertile, indicates a 56% share of the male factor in the occurrence of infertility [2]. In Poland, infertility affects 19% of couples, of which a male factor is responsible in 57% of cases [3].

This could be caused by intrinsic factors such as genetic or congenital disorders and cancer, but a decline in semen quality has also been observed in healthy men without any adverse medical history. In many cases, the etiology of diminished sperm quality generally remains unexplained. In addition to intrinsic factors, semen quality can also be affected by modifiable lifestyle behaviors, including diet, physical activity, comorbidities, environmental and occupational characteristics. For example, studies have suggested that specific nutritional factors can affect semen quality [4,5].

Studying dietary patterns (DPs) is a useful approach for describing the overall diet, including potential synergetic effects of food or nutrients, which are a crucial determinant of nutritional status linked with normal reproductive function. This approach uses collinearity between nutrients or food, examining the interrelation between the diet and its health effects [6]. To date, most observational or interventional nutrition studies have focused on analyzing the effect of single nutrients [7,8,9] or products [10,11,12,13] on semen quality. However, such studies have provided inconsistent results, probably due to neglecting the synergistic effect of multiple components of one’s diet [6]. Studies investigating the associations between DPs and semen quality suggest that some diets may have a protective effect on semen, while others reduce its quality [4]. Due to differences in study populations with high collinearity among lifestyle factors, the independent role of diet in influencing semen remains unclear.

Also, there is no large body of evidence that diet therapy may be effective in improving reproductive capacity; therefore, it might seem arguable to introduce changes in DPs or supplementation as a therapy to improve semen quality due to a lack of hard research endpoints in the form of the number of achieved pregnancies [4,14]. Moreover, these findings may have important public health implications, by taking semen quality as a predictor related to male health, morbidity and mortality [14,15]

Several studies have examined the association between dietary patterns and semen quality, but the findings have been inconclusive. There are a limited number of studies concerning the associations between diet and male infertility in the Polish population. Therefore, the present study aimed to investigate the association between DPs and the risk of abnormal semen quality parameters in Northern Eastern Polish men.

## 2. Materials and Methods

### 2.1. Participants

In this cross-sectional study sample was collected in 2014–2017 from men from Northern-Eastern Poland, who were attending the center of reproductive medicine in Olsztyn, Poland. Inclusion criteria were: men aged 20–55 years, absence of a specific clinical condition, voluntarily undergoing examinations, absence of acute and chronic disease conditions, and willingness to participate in the study confirmed by written consent. Exclusion criteria were: impairment or limitation of legal capacity, no consent for semen analysis, occurrence of chronic and reproductive tract diseases or diagnosed hormonal disorders.

**Figure 1:**
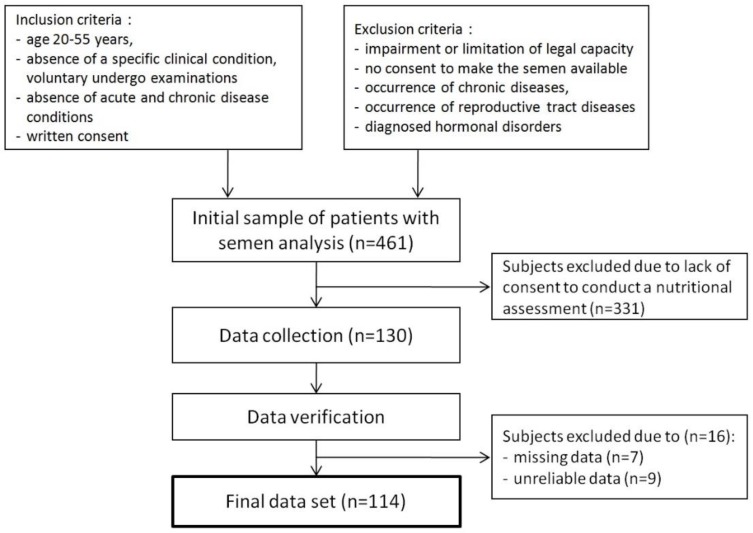
Flow chart of the study population.

Initially, the study population consisted of 461 men, who agreed to make available the results of the semen analysis. However, only 130 men were willing to participate in further stages of the study. During data verification, subjects were excluded due to missing (*n =* 7) and unreliable (*n =* 9) data. Finally, 114 (24.7%) men were enrolled in the study (Figure 1).

The study protocol was approved by the institutional review board of the Bioethical Committee of the Warmia-Mazury Medical Chamber in Olsztyn (No.9/2015). All participants gave written informed consent.

### 2.2. Dietary Data Collection

Dietary data was collected using a validated semi-quantitative 165-item food frequency questionnaire (FFQ) [16]. Men were asked about the frequency of food consumption within the year prior to involvement in the study. Options for frequency for each food item included seven categories: average amount per day, week, month or year and never, I do not know how often, I do not know if I ate. Food items from the FFQ were reduced to 23 food groups based on origin and similar nutrient content, and it was used for further analysis.

### 2.3. Semen Analysis

Semen samples were collected at the clinic by masturbation into a sterile plastic container. The samples were liquefied for 30 min in 37 °C before analysis. Macroscopic examination of semen was performed according to the 5th edition of WHO laboratory manual for the examination and processing of human semen [17]. Microscopic measurements of the sperm count, concentration, motility and morphology were determined with the use of computer-aided semen analysis (CASA). The basic components of the system were a bright field microscope (Olympus CX41, Tokyo, Japan), a digital camera to capture images (Olympus U-CMAD3), and a computer with software installed (SCA^®^Microptic S.L., Barcelona, Spain). The WHO [17] cut-off points were used to evaluate abnormal values of semen quality parameters (Table 1). All analyses were performed by an experienced technician.

### 2.4. Other Measurements

Body weight and height were taken using a digital scale and stadiometer, respectively, measured barefoot and in light clothing. Body mass index (BMI) was calculated (kg/m^2^), and WHO cut-off points were used to create the categories of weight status as underweight (<18.5 kg/m^2^), normal weight (18.5–24.99 kg/m^2^), overweight (25–29.99 kg/m^2^) and obese (>30 kg/m^2^) [18].

All participants completed self-administrated questionnaires, which collected information about economic status, educational level, place of residence, physical activity and sleep duration. Physical activity and sedentary time were assessed using the validated International Physical Activity Questionnaire (IPAQ)—long version [19]. Participants reported the average number of the duration (in minutes) and frequency (days) they spend on the following physical activities domains (during the week prior to the appointment): work-related, transport-related, domestic and gardening (yard), leisure-time. Physical activity (MET-hours/week) was calculated by multiplying the average total metabolic equivalents (MET) of walking, moderate and vigorous intensity activity (MET-min/week) across the four domains, summed and then divided by 60 min. Sedentary time (hour/week) was calculated as a sum of the average time per week spent on sitting at work and home, and while driving. Sleep duration was collected as a declared average time of sleeping (hours/day) during last year. Socioeconomic status was calculated by the information about the place of residence classified as village, <50 thousand inhabitants, 50–100 thousand inhabitants, >100 thousand inhabitants, economic status classified as below average, average, above average and educational level classified as basic and vocational, intermediate, high, and was presented in tertiles.

### 2.5. Statistical Analysis

Categorical variables were presented as a number (percentage) and continuous variables using mean (standard deviation). Normality of all continuous variables was evaluated using a Kolmogorov-Smirnov test. For comparison of categorical variables between groups, a χ^2^ test was used. To compare continuous variables, Student’s *t*-test and the Kruskal-Wallis test were used for variables with and without normal distribution, respectively. Presence of linear trends was assessed by Spearman correlation.

DPs were identified using factor analysis with principal component analysis with orthogonal (varimax normalized) rotation. Input variables included frequency of consumption of 23 food groups. Kaiser-Meyer-Olkin (KMO) value for food groups was 0.640 and Bartlett’s test had a significance of *p* < 0.001. Main factors (DPs) were determined by eigenvalues (>1.5) and a scree plot. The main factors were identified by factor load values (correlation coefficients) ≥0.40. Then, for each man, factor scores were calculated by regression approach and saved for each DP, representing the level of adherence to each specific pattern. Each of the patterns was divided into tertiles (bottom, middle, upper). Average consumption frequency of food items across the tertiles of dietary patterns are presented in Supplementary Materials (Table S1).

The association between DPs and semen quality parameters were evaluated using the logistic regression model. Odds ratios (ORs) and 95% confidence intervals (CIs) were calculated using nonlinear estimation with the Quasi-Newton and Rosenbrock Pattern Moves method. A reference group was made of study participants from the bottom tertile of dietary patterns with normal values of semen parameters (OR = 1). Two models were created: Crude—unadjusted; Adjusted—adjusted for BMI (kg/m2), physical activity (MET-h/week), sedentary time (h/day), sleep duration (h/day) (as continuous variables) and socioeconomic status (bottom, middle, upper tertiles) (as qualitative variables). OR significance was evaluated using the χ^2^ Wald test. *p*-value < 0.05 was considered significant in all tests. The statistical analysis was conducted with STATISTICA software (version 13.1 PL; StatSoft Inc.: Kraków, Poland).

## 3. Results

### 3.1. Dietary Patterns

Two DPs were derived: Pro-healthy and Western. Total variance explained was 29.3%. For each pattern, the explained variance was: 20.2% (Pro-healthy) and 9.1% (Western). Pro-healthy DP was described by daily frequency of consumption: fruits (*r* = 0.76), vegetables (*r* = 0.76), legumes (*r* = 0.66), soups (*r* = 0.55), mixed dishes (*r* = 0.47), whole-grain products (*r* = 0.43), juices (*r* = 0.43) and nuts (*r* = 0.42). Western DP was described by daily frequency of consumption: sweets and snacks (*r* = 0.71), processed meat (*r* = 0.59), animal fat (*r* = 0.57), refined grain products (*r* = 0.56), red meat (*r* = 0.53) potatoes (*r* = 0.50) and dairy products (*r* = 0.44) (Table 2).

### 3.2. Population Characteristics

Characteristics of the participants across tertiles of dietary patterns are shown in Table 3. The average age of study participants was 27.2 ± 7.6 years and their BMI was 24.7 ± 2.3 kg/m^2^. Participants from the upper tertile of Pro-healthy DP spent half as long sedentary than men from the bottom tertile (*p* < 0.05). Participants in the upper tertile of the Western DP were lower educated (*p* < 0.01), more physically active (*p* < 0.05), and predominantly had abnormal sperm progressive motility (*p* < 0.01).

### 3.3. Dietary Patterns and the Risk of Abnormal Semen Quality Parameters

There was no significant association between semen quality parameters in Crude and Adjusted models of Pro-healthy DP, and there was no trend for the risk of abnormal semen parameters. Significantly higher risk of abnormal progressive motility of semen was observed in the upper tertile of the Crude model of Western DP compare to the bottom tertile (OR: 3.86, 95% CI: 1.47–10.10). In the Adjusted model, the risk of abnormal progressive motility of semen was significantly higher in the middle (OR: 2.89, 95% CI: 1.03–8.09) and upper (OR: 7.78, 95% CI: 1.52–15.06) tertiles of Western DP. This DP was not associated with other semen quality parameters. In the Crude model of Western DP, a trend for the increased risk of abnormal progressive sperm motility was observed (*P*-trend = 0.021). In the Adjusted model, a trend for increased risk of abnormal total count (*P*-trend < 0.050), progressive motility (*P*-trend < 0.050) and morphology was found (*P*-trend < 0.050) (Table 4).

## 4. Discussion

The strong adherence to the Western DP was positively associated with the occurrence of abnormal sperm progressive motility. Moreover, this DP was related to the trends of abnormal count, progressive motility and morphology of sperm. In contrast, adherence to the Pro-healthy DP was not associated with semen quality parameters, regardless of the degree of adherence. Also, there was no effect after adjustment for potential confounders.

Our findings suggest that the high adherence to the Western DP (characterized by a frequent consumption of sweets and snacks, processed meat, animal fat, refined grain products, red meat, potatoes, and dairy products) was associated with a lower semen quality than that for men from the bottom tertile of this DP. This finding is consistent with the study by Eslamian et al. [11], who found that high adherence to Western DP was positively associated with the risk of asthenozoospermia [11] and decline in sperm concentration and morphology [20]. In contrast, studies by Gaskins et al. [21] and Oostingh et al. [22] did not find such association.

Animal products like red meat, processed meat and dairy could contribute to a decline in sperm quality [4]. These are great sources of protein and important micronutrients; however, they are also rich in saturated fatty acids (SFAs) and natural trans fatty acids (TFAs), which negatively influence sperm count and concentration [23,24]. In rodents, a diet rich in TFA and SFA led to number of reproductive dysfunctions like decreased serum testosterone level, testicular degeneration and stopping spermatogenesis [25,26]. Previous studies revealed that low-fat dairy intake was positively related to progressive motility and sperm concentrations [27], while high intake of full-fat dairy has been linked to lower progressive motility and morphology among subfertile men [28]. Also, greater intake of dairy was associated with oligoasthenoteratospermia and asthenospermia in men from infertile couples [10,12]. Nonetheless, we can only speculate on the potential mechanisms of SFAs and TFAs on the human reproductive system, and the effects of their high intake should be examined in the future studies [4]. Animal products also contain numerous natural substances and additives which are added during the production process, which may negatively affect semen quality [29]. A large part of them is highly lipophilic and can accumulate in the high-fat products like red meat and dairy [4]. Presence of preservative agents or hormonal residues, like xenobiotics, included xenoestrogens, anabolic steroids, other pregnancy hormones and environmental chemicals, may incorporate into sperm membrane and as a result lower semen motility [29]; however, their potential impact on the male reproductive system is still lacking sufficient evidence [12,30].

Other foods which negatively affected semen quality and were components of Western DPs were sweets and snacks, and refined grains. Liu et al. [20] reported that ‘Highly sweet snack & sugar-sweetened drinks’ and ‘High-carbohydrate food’ DPs were associated with lower sperm concentration and increased prevalence of abnormal total sperm motility and progressive motility, respectively. Similarly, Eslamian et al. [10] pointed out that sweets consumption was positively associated with the risk of asthenozoospermia. Increase of insulin resistance may cause an increase of the oxidative stress [31], which negatively influences semen quality (Mendiola et al. 2010). Also, the action of hypothalamic-pituitary-testis axis responsible for sperm production can be disrupted by glucose and insulin [32], whereas the high intake of fiber may lower a glycemic load of products or meals and, through binding directly to unconjugated estrogens, may reduce their plasma level, leading to declining the risk of asthenozoospermia [11].

To our surprise, in the present study, the Pro-healthy DP (characterized by a frequent consumption of fruits, vegetables, legumes, soups, mixed dishes, whole-grain products, juices and nuts) was not associated with any of the semen parameters. These findings stand in contrast to an increasing number of research findings indicating a consistent relation between intake of healthy foods and diet, and semen quality parameters as proxy measures of male fertility. Similarly, Liu CY et al. [20] did not find any associations between Healthy DP (characterized by a frequent consumption of light-color vegetables, dark-color vegetables and fruits) and semen parameters. A possible explanation is that the consumption of conventionally grown fruits and vegetables may be a major source of cumulative pollutants and pesticides, which cause lower semen quality [13] The pesticides and insecticides contained in plants may have a stronger impact on the deterioration of the quality of the semen than benefits of micronutrients, vitamins and antioxidants in them. [33]. It could be an important datum on which should be focused on, in the context of the varied quality, manufacturing methods and availability of food nowadays. It also could relate to high-frequency consumption of legumes, especially soy, rich in isoflavones, which may be supposed to lower the sperm concentration [34], but its intake is unrelated to clinical treatment outcomes [35]. However, the majority of studies have shown a positive relationship between healthy diet, foods or nutrients and the quality of semen. Gaskins et al. [21] found that Prudent DP was related to higher positive sperm motility; Jurewicz et al. [36,37] declared that Prudent DP decrease the DNA fragmentation index, disomy of chromosomes XX and 21, and was positively related to sperm concentration and testosterone level; Oostingh et al. [22] and Karayiannis et al. [38] found that strong adherence to Healthy DP and Mediterranean diet was significantly associated with higher sperm concentration, total sperm count and progressive motility. Eslamian et al. [10,39] pointed out that high intake of fruits and vegetables, dark green vegetables, skim milk, poultry and seafood and diet rich in vitamin E, vitamin D, vitamin C, selenium, zinc and PUFAs was significantly associated with lower risk of asthenozoospermia, most likely due to its antioxidative properties It is estimated that in 30–80% cases, impairment of male fertility can be caused by damage to sperm as a result of oxidative stress [9]. Fruit and vegetables are rich in antioxidants (beta-carotene, vitamin E and vitamin C and polyphenols), fiber, folate, vitamin B6, which play an important role in ensuring the quality of semen. Their possible effect may consist in reducing oxidative stress and chronic inflammation while improving the function of semen, reduces the amount of damaged DNA, participating in steroid hormone synthesis, inhibiting spermaglutination and most probably protecting against the toxic effect of heavy metals [7,40,41,42,43]. However, only a few small randomized controlled trials have suggested that subfertile men treated with antioxidant supplementation may improve live birth rates and clinical pregnancy, but there was lack of evidence of increased risk of miscarriage and on other adverse effects [9]. It needs to be highlighted that semen quality and risk factors for its decreasing is a poor predictor of fertility and male reproductive performance [8,23,34,35].

Studies on the relationship between nutritional status, lifestyle factors, like physical activity, sedentary time, sleep duration and socioeconomic status, which were used in our study as adjustment factors, and semen quality are scarce and inconsistent, and their role and potential mechanisms in male infertility are still unclear. In our study, BMI did not differ between dietary patterns. We expected elevated BMI especially in middle and upper tertiles of western DP, but a multitude of confounders may bias this result, like increasing physical activity, and differently correlated sedentary time or sleep duration. Both vigorous and light physical activity, as well as overall physical activity and also sedentary time, including television watching time, were linked or could negatively affect semen parameters [5,44] and reproductive hormones [44]. However, some studies did not find these associations [45] or, moreover, presented no association with reproductive outcomes [5]. There is a limited number of studies on sleep duration and semen quality, but they underline that restricted or excessive sleep may negatively affect semen quality [46,47]. Socioeconomic status impact on male reproductive health is largely unknown and required further research. Perhaps the effect of non-diet lifestyle factors on semen could explain the null relation between Pro-healthy DP and semen quality parameters in our study.

There were some limitations in the current study. First, although the validated FFQ was used, it was not possible to entirely exclude measurement errors and recall bias in this study. Secondly, we were not able to examine a representative sample of the general male population, due to lack of consent to conduct a nutritional assessment, but participants were heterogeneous in their semen parameters. Thirdly, only one semen sample was taken from each participant, but they were collected and analyzed in one laboratory, which increases the internal validity of the data. Finally, the nature of a cross-sectional study did not allow determining a possible link for observed relations. It may only indicate potential interrelations, providing the basis for designing future interventions.

The strengths of our study are, first, the principal component analysis used in our study is the gold standard for identifying DPs. This method makes it possible to reflect the real dietary behaviors present in a population by high correlations of food consumption with the factor, leading to increased power to detect diet-disease relationships [6,11]. Secondly, the FFQ we used was validated on a Polish population and is a comprehensive method that allows a detailed description of dietary behaviors. Thirdly, we excluded participants with overestimated consumption. Another advantage of our study was that frequency of food consumption was controlled for potential confounders such as socioeconomic status, BMI, energy expenditure on physical activity, sedentary time and sleep duration, which may influence the dietary behaviors.

Finally, although several studies have explored the association between dietary patterns and semen quality, only few have reported results from adults from Central or Eastern Europe. The current paper fills this important gap by exploring the link between dietary patterns, including the Western dietary pattern, and the risk of semen quality in North-Eastern Poland.

## 5. Conclusions

The present study provides interesting insights into the harmful effect of adherence to the western dietary pattern and the risk of abnormal sperm progressive motility and a trend of an abnormal count, progressive motility and morphology of semen. Perhaps the associations between healthy diet and semen quality could be revealed if a larger sample were studied. Further longitudinal studies are needed to clarify this relationship; in particular, well-designed randomized control trials based on dietary patterns are needed. Examination of these interactions may contribute to the development of more efficient preventive and interventional programs adjusted to the specificity of nutritional and health problems in paternity planning.

## Figures and Tables

**Table 1:** Cut-off points used to evaluate abnormal values of semen quality parameters [17].

Semen Parameters	Cut-Off Points
Sperm concentration	<15 × 10^6^/ml
Sperm count	<39 × 10^6^/ejaculate
Total motility	<40%
Progressive motility	<32%
Morphology	<4% of normal forms

**Table 2:** Factor loadings of identified dietary patterns.

Variables	Dietary Patterns	
	Pro-Healthy	Western
Fruits	**0.76**	0.11
Vegetables	**0.76**	0.12
Legumes	**0.66**	0.32
Soups	**0.55**	0.25
Mixed dishes	**0.47**	0.28
Whole-grain products	**0.43**	−0.24
Juices	**0.43**	0.39
Nuts	**0.42**	0.09
Sweets and snacks	−0.04	**0.71**
Processed meat	0.07	**0.59**
Animal fat	0.09	**0.57**
Refined grain products	0.24	**0.56**
Red meat	0.04	**0.53**
Potatoes	0.30	**0.50**
Dairy products	0.20	**0.44**
Fish and seafood	0.39	−0.03
Sweetened fruit products	0.39	0.37
Eggs	0.38	−0.04
Plant oils	−0.01	0.38
Coffee and tea	0.03	0.38
Beverages	−0.00	0.32
Alcohol drinks	−0.24	0.29
Poultry	0.20	−0.00
Variance explained (%)	**20.2**	**9.1**

**Highlighted** are factor loadings of ≥0.4 included in identified factors. Total explained variance was 29.3%. KMO = 0.672; Bartlet’s test < 0.001.

**Table 3:** Characteristics of the participants across tertiles of dietary patterns.

Variables	Total Sample	Pro-Healthy Dietary Pattern			*p* ^1^	Western Dietary Pattern			*p* ^1^
		Bottom	Middle	Upper		Bottom	Middle	Upper	
*n*	114	37	38	39		38	37	39	
Factor scores of dietary patterns		−2.57 to −0.48	> −048 to 0.20	>0.20 to 4.42		−2.06 to −0.51	> −0.51 to 0.30	>0.30 to 3.26	
Age (years)	27.2 ± 7.6	27.1 ± 7.5	27.6 ± 7.8	26.9 ± 7.6	0.718	28.4 ± 8.9	26.3 ± 6.6	26.9 ± 7.0	0.894
Place of residence					0.299				0.197
Village and city < 50 thousand citizens	75 (65.8)	25 (67.6)	24 (31.2)	26 (66.7)		23 (60.5)	27 (72.9)	25 (64.1)	
City 50–100 thousand citizens	19 (16.7)	3 (8.1)	7 (18.4)	9 (23.1)		6 (15.8)	3 (8.1)	10 (25.6)	
City > 100 thousand citizens	20 (17.5)	9 (24.3)	7 (18.4)	4 (10.2)		9 (23.7)	7 (18.9)	4 (10.3)	
Economic status					0.826				0.531
Below average	2 (1.8)	1 (2.7)	0 (0.0)	1 (2.6)		0 (0.0)	1 (2.7)	1 (2.6)	
Average	95 (83.3)	31 (83.8)	31 (81.6)	33 (84.6)		34 (89.5)	28 (75.7)	33 (84.6)	
Above average	17 (14.9)	5 (13.5)	7 (18.4)	5 (12.8)		4 (10.5)	8 (21.6)	5 (12.8)	
Educational level					0.983				0.006
Basic and vocational	16 (14.0)	6 (16.2)	5 (13.2)	5 (12.8)		2 (5.3)	4 (10.8)	10 (25.6)	
Intermediate	54 (47.4)	18 (48.7)	18 (47.4)	18 (46.2)		14 (36.8)	19 (51.4)	21 (53.9)	
High	44 (38.6)	13 (35.1)	15 (39.5)	16 (41.0)		22 (57.9)	14 (37.8)	8 (20.5)	
Socioeconomic status (tertiles)					0.827				0.205
Bottom	32 (28.1)	12 (32.4)	11 (28.9)	9 (23.1)		6 (15.8)	11 (29.7)	15 (38.5)	
Middle	34 (29.8)	9 (24.3)	11 (28.9)	14 (35.9)		12 (31.6)	10 (27.1)	12 (30.8)	
Upper	48 (41.1)	16 (43.3)	16 (42.1)	16 (41.0)		20 (52.6)	16 (43.2)	12 (30.8)	
BMI (kg/m^2^)	24.7 ± 2.3	24.7 ± 2.7	24.8 ± 2.1	24.7 ± 2.2	0.953	24.8 ± 1.9	24.7 ± 2.6	24.6 ± 2.4	0.750
BMI < 25 kg/m^2^	67 (58.8)	21 (56.8)	22 (57.9)	24 (61.5)	0.906	20 (52.6)	25 (67.6)	22 (56.4)	0.394
BMI ≥ 25 kg/m^2^	47 (41.2)	16 (43.2)	16 (42.1)	15 (38.5)		18 (47.4)	12 (32.4)	17 (43.6)	
Physical activity (MET-h/week)	135.2 ± 78.9	122.2 ± 74.3	138.2 ± 79.4	144.6 ± 83.1	0.574	120.2 ± 84.0	121.0 ± 65.2	163.3 ± 80.1	0.014
Sedentary time (h/day)	3.0 ± 2.5	4.0 ± 3.1	2.7 ± 2.1	2.3 ± 1.7	0.020	2.5 ± 1.8	3.0 ± 2.6	3.5 ± 2.8	0.360
Sleep duration (h/day)	7.1 ± 1.1	7.1 ± 1.2	6.9 ± 1.0	7.2 ± 1.1	0.572	7.1 ± 1.2	7.0 ± 1.2	7.3 ± 1.0	0.492
Sperm concentration (10^6^/ml)	53.9 ± 65.4	52.5 ± 51.4	50.9 ± 59.4	58.0 ± 82.2	0.702	62.5 ± 68.9	57.0 ± 59.8	42.4 ± 67.1	0.423
Abnormal sperm concentration ^2^	37 (32.5)	10 (27.0)	12 (31.6)	15 (38.5)	0.562	9 (23.7)	13 (35.1)	15 (38.5)	0.351
Sperm count (10^6^/ejaculate)	178.7 ± 225.5	173.0 ± 185.7	169.1 ± 187.2	193.3 ± 290.1	0.686	226.1 ± 252.9	180.6 ± 196.9	130.6 ± 218.2	0.274
Abnormal sperm count ^2^	32 (28.1)	10 (27.0)	8 (21.1)	14 (35.9)	0.345	8 (21.1)	11 (29.7)	13 (33.3)	0.469
Total motility (%)	48.0 ± 16.3	50.5 ± 16.1	47.1 ± 15.9	46.6 ± 16.9	0.611	51.7 ± 15.2	45.2 ± 17.7	47.1 ± 15.6	0.239
Abnormal total motility ^2^	39 (34.2)	12 (32.4)	12 (31.6)	15 (38.5)	0.786	9 (23.7)	15 (40.5)	15 (38.5)	0.241
Progressive motility (%)	30.4 ± 14.0	32.6 ± 13.6	29.8 ± 13.7	28.9 ± 14.8	0.596	34.6 ± 13.8	28.6 ± 14.7	28.0 ± 12.9	0.055
Abnormal progressive motility ^2^	63 (55,3)	20 (54.1)	22 (57.9)	21 (53.9)	0.923	14 (36.8)	22 (59.5)	27 (69.2)	0.014
Morphology (% of normal forms)	7.8 ± 5.9	7.7 ± 5.6	7.4 ± 6.0	8.1 ± 6.4	0.856	7.9 ± 6.3	7.8 ± 6.2	7.5 ± 5.4	0.989
Abnormal morphology ^2^	28 (25.6)	8 (21.6)	9 (23.7)	11 (28.2)	0.791	9 (23.7)	8 (21.6)	11 (28.2)	0.791

BMI: body mass index, MET: metabolic equivalent of tusk. Data are presented as mean ± SD for continuous variables and *n* (%) for categorical variables. ^1^ *p*-values for continuous variables were derived from Kruskal-Wallis test or Student’s *t*-test and for categorical variables were derived from χ^2^ test or Spearman’s rho test. ^2^ compared to group with normal semen parameters.

**Table 4:** Odds ratios (95% confidence intervals) for abnormal semen quality across tertiles of dietary patterns.

Abnormal Semen Quality Parameters	Dietary Patterns							
	Pro-Healthy				Western			
	Bottom	Middle	Upper	*P*-trend	Bottom	Middle	Upper	*P*-trend
*n*	37	38	39		38	37	39	
Total count								
Crude	ref	0.72 (0.24; 2.13)	1.51 (0.56; 4.08)	0.561	ref	1.58 (0.54; 4.62)	1.88 (0.66; 5.32)	0.116
Adjusted	ref	0.66 (0.21; 2.09)	1.49 (0.50; 4.41)	0.600	ref	1.43 (0.47; 4.36)	1.86 (0.58; 6.01)	<0.050
Sperm concentration								
Crude	ref	1.25 (0.45; 3.44)	1.68 (0.63; 4.53)	0.097	ref	1.75 (0.63; 4.86)	2.01 (0.74; 5.50)	0.134
Adjusted	ref	1.07 (0.37; 3.10)	1.82 (0.62; 5.33)	0.284	ref	1.72 (0.60; 4.93)	2.01 (0.65; 6.20)	0.153
Progressive motility								
Crude	ref	1.17 (0.44; 3.13)	0.99 (0.40; 2.45)	0.969	ref	2.51 (0.98; 6.47)	3.86 (1.47; 10.10) ***	<0.050
Adjusted	ref	1.46 (0.54; 3.97)	1.21 (0.43; 3.34)	0.699	ref	2.89 (1.03; 8.09) *	4.78 (1.52; 15.06) ***	<0.050
Total motility								
Crude	ref	0.96 (0.36; 2.58)	1.30 (0.50; 3.40)	0.402	ref	2.20 (0.80; 6.04)	2.01 (0.74; 5.50)	0.427
Adjusted	ref	1.30 (0.44; 3.82)	1.46 (0.50; 4.29)	0.111	ref	2.31 (0.76; 7.01)	2.43 (0.76; 7.75)	0.285
Morphology								
Crude	ref	1.13 (0.37; 3.38)	1.42 (0.49; 4.13)	0.138	ref	0.89 (0.30; 2.67)	1.26 (0.45; 3.58)	0.154
Adjusted	ref	1.22 (0.37; 4.07)	1.32 (0.41; 4.23)	0.136	ref	1.11 (0.34; 3.57)	1.22 (0.37; 4.01)	<0.050
TMSC								
Crude	ref	0.84 (0.49; 2.41)	1.88 (0.70; 5.02)	0.425	ref	1.75 (0.63; 4.86)	1.61 (0.58; 4.46)	0.446
Adjusted	ref	0.95 (0.29; 3.06)	1.76 (0.61; 5.07)	0.368	ref	1.60 (0.55; 4.66)	1.56 (0.49; 5.01)	0.371

The reference group for analyzed parameters were men with normal semen quality values according to WHO [17]. Crude—unadjusted model, Adjusted—model adjusted for BMI: body mass index (kg/m^2^), physical activity (MET-h/week), sedentary time (h/day), sleep duration (h/day) (all as continuous variables) and socioeconomic status (as qualitative variables). * *p* < 0.05; *** *p* < 0.01.

## Supplementary Materials

The following are available online at http://www.mdpi.com/2072-6643/10/9/1162/s1, Table S1: Mean with standard deviation (SD) for dietary characteristics across tertiles of dietary patterns (*n* 114).
