# Supplementary material for: Dietary Patterns and Poor Semen Quality Risk in Men: A Cross-Sectional Study
Source: Nutrients. 2018 Aug 24;10(9):1162. doi: 10.3390/nu10091162 (PMC6165100; doi:10.3390/nu10091162)
Supplement: Supplementary file 1 [file nutrients-10-01162-s001.pdf]

Table S1. Mean with standard deviation (SD) for dietary characteristics across tertiles of dietary patterns (*n* 114)

Dietary items	Total	Pro-healthy DP			*p*	Western DP			*p*
		bottom	middle	upper		bottom	middle	upper	
n		37	38	39		38	37	39	
Fruits	1.62 (1.13)	0.79 (0.54)	1.52 (0.85)	2.49 (1.18)	**<0.001**	1.53 (0.97)	1.35 (1.18)	1.96 (1.18)	0.034
Vegetables	3.01 (2.12)	1.63 (1.01)	2.54 (1.30)	4.79 (2.32)	**<0.001**	2.34 (1.72)	3.29 (2.45)	3.42 (2.02)	0.019
Legumes	0.23 (0.24)	0.10 (0.10)	0.20 (0.20)	0.39 (0.30)	**<0.001**	0.13 (0.13)	0.26 (0.29)	0.31 (0.26)	0.001
Soups	0.46 (0.35)	0.27 (0.22)	0.45 (0.28)	0.65 (0.40)	**<0.001**	0.32 (0.28)	0.47 (0.40)	0.58 (0.32)	0.001
Mixed dishes	0.55 (0.36)	0.42 (0.30)	0.47 (0.28)	0.76 (0.40)	**<0.001**	0.39 (0.29)	0.65 (0.41)	0.62 (0.33)	0.001
Whole-grain products	0.73 (0.86)	0.34 (0.46)	0.80 (0.89)	1.02 (1.00)	**<0.001**	0.98 (0.93)	0.71 (0.83)	0.50 (0.76)	0.003
Juices	1.03 (0.89)	0.66 (0.88)	0.77 (0.56)	1.64 (0.86)	**<0.001**	0.68 (0.57)	0.99 (0.70)	1.41 (1.15)	0.003
Nuts	0.20 (0.29)	0.09 (0.08)	0.19 (0.18)	0.33 (0.42)	**0.003**	0.16 (0.15)	0.22 (0.30)	0.22 (0.37)	0.921
Sweets and snacks	2.77 (1.89)	3.04 (2.37)	2.44 (1.67)	2.85 (1.56)	0.428	1.44 (0.90)	2.52 (1.41)	4.32 (1.92)	**<0.001**
Processed meat	4.58 (2.98)	4.65 (3.14)	4.08 (2.63)	5.02 (3.14)	0.437	2.66 (1.48)	4.50 (2.13)	6.54 (3.52)	**<0.001**
Animal fat	4.46 (3.13)	3.90 (3.70)	4.95 (2.78)	4.51 (2.82)	0.130	2.61 (2.14)	4.55 (2.79)	6.17 (3.28)	**<0.001**
Refined grain products	2.69 (1.74)	2.62 (1.69)	2.41 (1.14)	3.04 (2.20)	0.828	1.52 (0.72)	2.91 (1.57)	3.63 (1.96)	**<0.001**
Red meat	0.59 (0.64)	0.58 (0.54)	0.47 (0.31)	0.73 (0.91)	0.390	0.34 (0.30)	0.50 (0.33)	0.93 (0.93)	**<0.001**
Potatoes	0.83 (0.55)	0.68 (0.41)	0.65 (0.36)	1.13 (0.68)	<0.001	0.50 (0.30)	0.81 (0.48)	1.16 (0.60)	**<0.001**
Dairy products	3.57 (2.07)	3.69 (2.42)	3.10 (1.76)	3.91 (1.96)	0.193	2.75 (1.50)	3.54 (2.07)	4.39 (2.27)	**0.003**
Fish and seafood	0.34 (0.39)	0.18 (0.18)	0.34 (0.40)	0.50 (0.46)	0.001	0.32 (0.30)	0.40 (0.48)	0.30 (0.37)	0.682
Sweetened fruit products	0.39 (0.48)	0.25 (0.39)	0.30 (0.38)	0.62 (0.57)	<0.001	0.18 (0.23)	0.38 (0.45)	0.62 (0.59)	<0.001
Eggs	0.36 (0.36)	0.22 (0.16)	0.31 (0.23)	0.53 (0.51)	<0.001	0.35 (0.47)	0.37 (0.25)	0.35 (0.34)	0.410
Plant oils	0.89 (0.94)	0.88 (1.29)	0.85 (0.71)	0.92 (0.76)	0.503	0.66 (0.49)	0.72 (0.63)	1.26 (1.35)	0.057
Coffee and tea	1.92 (1.41)	2.02 (1.52)	1.84 (1.26)	1.89 (1.46)	0.916	1.31 (0.68)	1.92 (1.43)	2.51 (1.66)	0.003
Beverages	0.60 (0.68)	0.60 (0.73)	0.66 (0.78)	0.53 (0.49)	0.995	0.40 (0.43)	0.59 (0.45)	0.80 (0.95)	0.166
Alcohol drinks	0.50 (0.47)	0.63 (0.68)	0.43 (0.36)	0.43 (0.28)	0.765	0.31 (0.24)	0.49 (0.35)	0.68 (0.65)	0.005
Poultry	0.44 (0.52)	0.29 (0.24)	0.40 (0.38)	0.62 (0.74)	0.007	0.50 (0.66)	0.33 (0.23)	0.48 (0.56)	0.643

DP: dietary pattern, n: number. Data are presented as mean (SD). *p*-values were derived from Kruskal–Wallis test or Student's *t*-test
